# Study on 3D Characteristics of Pores in Bimodal SiC_p_ Preforms Using X-Ray Micro-Computed Tomography

**DOI:** 10.3390/ma19183832

**Published:** 2026-09-09

**Authors:** Ruizhe Liu, Yuchen Feng, Hu Xu, Xiaoyu Wang, Tao Wen

**Affiliations:** School of Intelligent Manufacturing, Guangzhou Maritime University, Guangzhou 510725, China

**Keywords:** bimodal SiC_p_ preforms, particle fraction, 3D μ-CT, pore characteristics

## Abstract

**Highlights:**

- X-ray μ-CT was used to extract 3D pore networks in bimodal SiC_p_ preforms.
- Areal porosity was minimized at a 67% coarse fraction while pore homogeneity declined.
- Particle contact and fracture caused deviations from empirical surface areas.
- Higher coarse fractions decreased pore numbers but increased their average sizes.

**Abstract:**

Particle-reinforced metal matrix composites, wherein preform pore structure dominates liquid infiltration behavior and final composite quality, are essential for high-performance industries. Conventional empirical models predict pore characteristics for bimodal preforms based on ideal particle stacking assumptions yet ignore real compression-induced microstructural changes including particle contact compaction and particle fracture, yielding systematic deviations from actual pore characteristics. This study adopted high-resolution 3D X-ray micro-computed tomography (μ-CT) to quantify such discrepancies for bimodal SiC_p_ preforms across six coarse-to-fine particle ratios (0–100%). Three-dimensional pore network models were extracted to quantify key characteristics including areal porosity, surface area, and pore/throat dimensions. The results demonstrated that the average areal porosity fell to a minimum at a 67% coarse fraction then rose, while the pore distribution homogeneity steadily declined. Additionally, μ-CT measurements revealed that particle contact compactness reduced the particle surface area per unit volume at coarse fractions below 25% whereas particle fracture increased it at fractions above 25%, deviating significantly from empirical predictions. Larger coarse particle fractions reduced pore/throat quantities but increased their average size and volume. Beyond using established pore network extraction, this work distinguishes these two competing micro mechanisms and provides reasonable datasets to support bimodal preform optimization for composite manufacturing.

## 1. Introduction

Particle-reinforced metal matrix composites have attracted extensive attention in the aerospace, advanced equipment and electronic industries owing to their superior comprehensive mechanical and thermal properties [1,2,3]. Liquid-phase infiltration, which involves the preparation of porous preforms and subsequent molten metal infiltration, is one of the most efficient and widely adopted techniques for manufacturing such composites [4,5,6]. The pore characteristics of preforms significantly affect the infiltration process and further determine the final microstructures and properties of composites [7,8,9]. To design pore structures, preforms can be prepared using bimodal particles with different coarse-to-fine particle ratios. Conventional methods for evaluating pore characteristics include Archimedes’ method and empirical equations [10,11,12,13]. Empirical equations are commonly used to calculate the surface area per unit volume of particles in bimodal SiC_p_ preforms [14,15,16]. However, these conventional analytical approaches are unable to account for realistic physical changes during the compression molding process, such as the particle characteristics and contact compactness. Neglecting these actual characteristics inevitably leads to deviations in the calculation of particle surface areas and limits the understanding of preform structures.

Excessive reliance on simplified empirical models creates a noticeable analytical gap, failing to reflect the complex nature of realistic preforms and limiting the precise optimization of composite manufacturing. To overcome the limitations of conventional modeling methods, high-resolution 3D X-ray μ-CT has emerged as an advanced non-destructive testing technique for the characterization and analysis of material microstructures [17,18,19,20]. Unlike conventional tests that only provide macroscopic parameters, μ-CT enables visualized and hierarchical reconstruction of internal pore structures and allows accurate measurement of 2D and 3D characteristics [21,22,23]. The realistic pore structure obtained via 3D μ-CT scanning enables the extraction of 3D pore network models using algorithms such as the Maximal Ball (MB) algorithm [24,25,26]. This algorithm enables the quantitative determination of key characteristics of pores and throats, including their radius, volume, length, and coordination number, supporting comprehensive quantitative analysis of pore 3D characteristics [27,28].

To address the urgent demand for transition from conventional empirical analysis to realistic 3D structural analysis, this study utilized high-resolution 3D X-ray μ-CT to investigate the realistic 3D characteristics of pores in bimodal SiC_p_ preforms with different coarse-to-fine particle ratios. Six groups of preforms with coarse particle fractions ranging from 0% to 100% were fabricated and inspected. Based on the X-ray μ-CT detection results, the 3D pore network models were extracted to quantitatively analyze pore characteristics, especially areal porosity distributions, particle surface areas per unit volume and the sizes of pores and throats. Through a comparative analysis between μ-CT experimental data and empirical calculation results, this study evaluated the effects of particle contact compactness and particle fracture on the internal structures of preforms. This work reveals the intrinsic link between bimodal particle ratios and pore 3D characteristics and provides experimental support for optimizing the manufacturing and performance of bimodal porous preforms.

## 2. Materials and Methods

### 2.1. Preparation of Bimodal SiC_p_ Preforms

**Figure 1:**
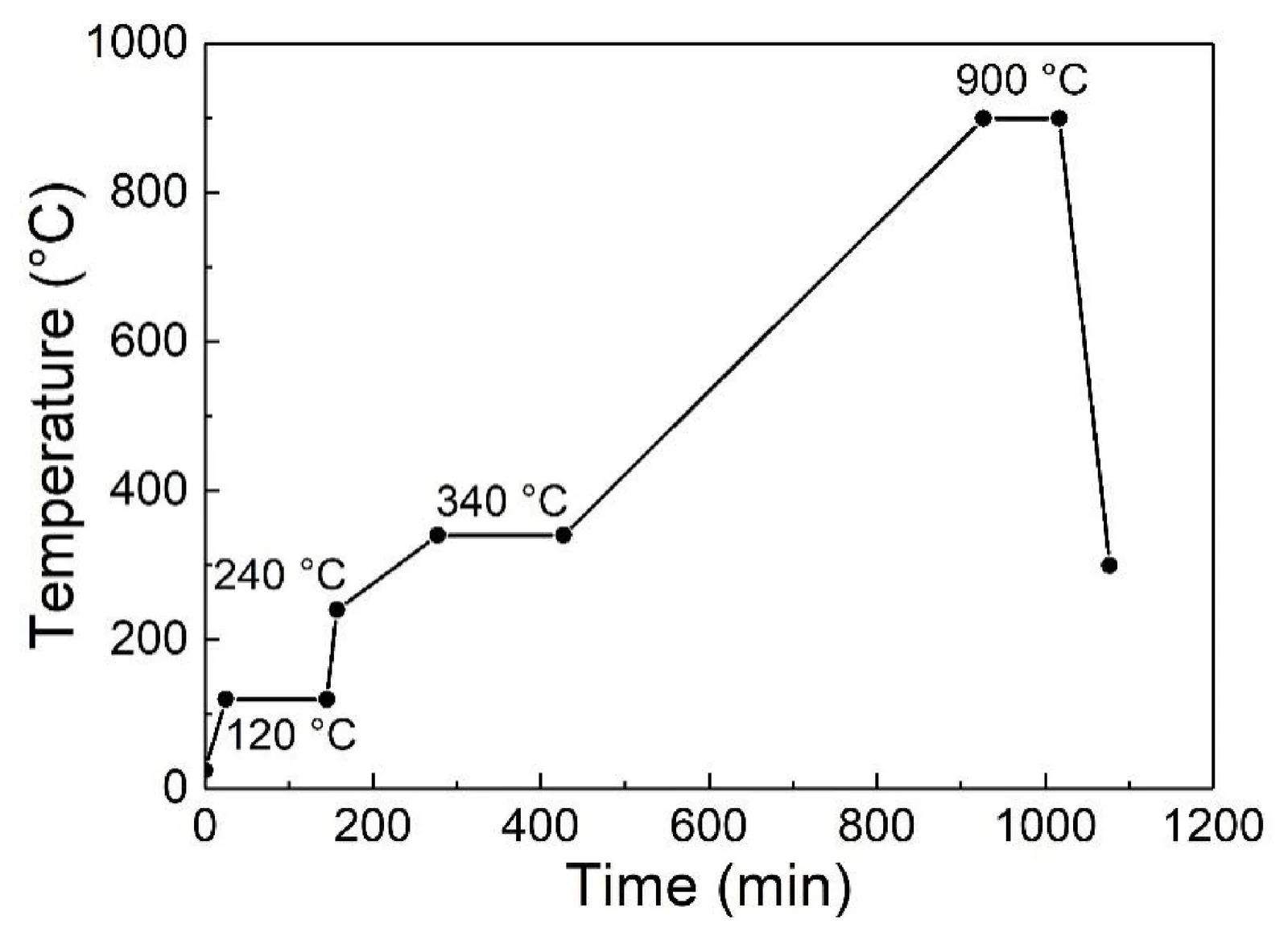
High-temperature sintering process of bimodal SiC_p_ preforms.

In this experiment, SiC particles (Zhongnuo Advanced Material Technology Co., Ltd. (Beijing, China)) with purity exceeding 99.7% and particle sizes of 170 or 20 μm were selected as reinforced particles to study the effects of different coarse-to-fine particle ratios on the pore characteristics of preforms. Silica sol was chosen as the binder. Based on the design by Molina [13,14,15,16], six types of preforms with different coarse (170 μm) SiC particle fractions (0%, 25%, 50%, 67%, 75% and 100%) were prepared. Coarse and fine (20 μm) SiC particles in the specified ratios were put into a powder mixer and mixed for 12 h to obtain homogeneous mixed powders. Then binder equivalent to 10% of the mixed powder volume was added and thoroughly mixed. The mixtures were put into a hydraulic press (HJS32-100, Huzhou Machine Tool Works Co., Ltd. (Huzhou, China)), and uniaxial pressure of 100 MPa was applied for 2 min to produce 30 mm diameter preforms. Six types of bimodal SiC_p_ preforms were prepared according to the high-temperature sintering curve shown in Figure 1 (c-0%, c-25%, c-50%, c-67%, c-75% and c-100%; c-0% means that the preform consisted entirely of fine particles, c-25% means that the preform contained 25% coarse particles, and so on). Three samples were prepared for each type of preform to verify accuracy. The porosities of the bimodal SiC_p_ preforms were measured with Archimedes’ method (as described in our previous study [27]).

### 2.2. Detection with 3D X-Ray μ-CT

Cubic samples of size 2 × 2 × 2 mm^3^ were first cut from the preforms, then slowly ground down to 1 × 1 × 1 mm^3^ using metallographic abrasive papers, and finally analyzed using a high-resolution (maximum spatial resolution: 0.5 μm; uncertainty: 1.2–1.5 times the voxel size) 3D X-ray μ-CT device (nanoVoxel-3502E, Tianjin Sanying Precision Instrument Co., Ltd. (Tianjin, China)). During the scanning experiment, the voltage and current were set to 70 kV and 145 μA, respectively, and the isotropic voxel used for scan reconstruction was 1 μm. The samples were rotated in a circle with an angular step size of 0.5°/min for a series of X-ray tomography raw images with 1024 × 1024 pixels. Considering both the scanning accuracy of μ-CT and data processing efficiency, a 600 × 600 × 600 μm^3^ volume of interest (VOI) was extracted from the defect-free center of each CT sample for testing and analysis, 200 μm away from any sample boundary to minimize the boundary effects. Then, the cross sections along the *Z*-axis of the samples were reconstructed with μ-CT. In order to quantitatively evaluate the effects of coarse-to-fine particle ratios on the homogeneity of pore distributions in the preforms, the areal porosity fractions and standard deviation values of samples (STDEV.S) of *Z*-axis cross sections of the preforms were analyzed. The 3D morphologies of the preforms were reconstructed using Volume Graphics software (VG Studio Max 2.2), and the 3D characteristics of the pores were analyzed.

### 2.3. Inspection of 3D Pore Networks of Preforms

Three-dimensional pore networks with equivalent geometric topologies extracted according to the reconstructed morphologies are useful in quantitative analyses of preform characteristics. The 3D pore network extraction method is based on the MB algorithm. In this algorithm, MBs are defined as the inscribed balls between particles, pores are defined as the neighboring and overlapped MBs, and throats are regarded as the small-sized MBs connecting adjacent pores. This algorithm allows for the determination of key characteristics of pores and throats, including their effective radius, volume, length, coordination number and shape factor, thereby enabling calculation of the preform porosities and further statistical analysis of the average values and frequency distributions of pore and throat characteristics.

## 3. Results and Discussion

### 3.1. 2D Cross Section Characteristics of Preforms

**Figure 2:**
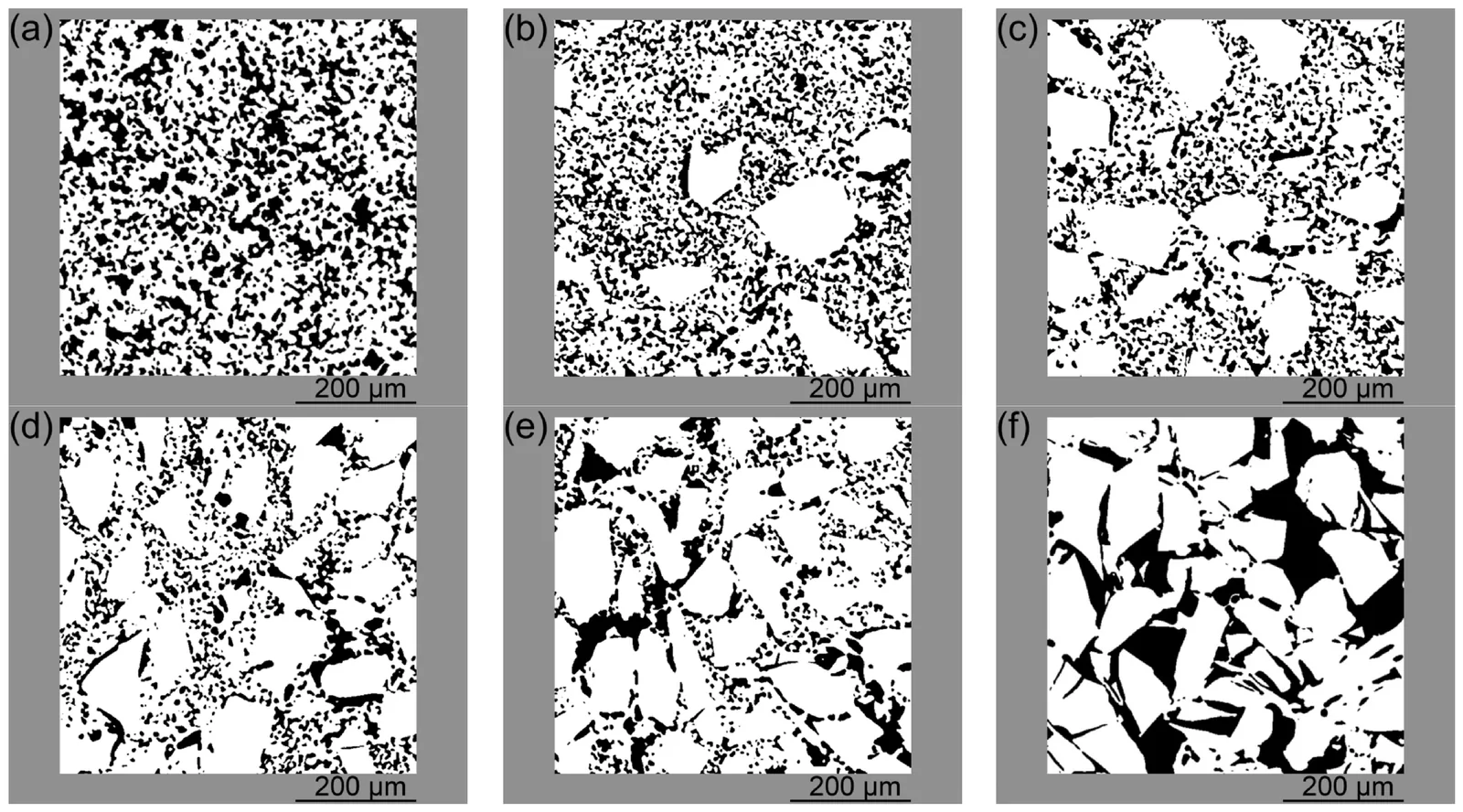
Morphologies of *Z*-axis cross sections of bimodal SiC_p_ preforms: (**a**) c-0%, (**b**) c-25%, (**c**) c-50%, (**d**) c-67%, (**e**) c-75%, and (**f**) c-100%.

Figure 2 presents the morphologies of *Z*-axis cross sections of bimodal SiC_p_ preforms with different coarse-to-fine particle ratios, where the particles are shown in white and the pores are shown in black. As shown in Figure 2a, the c-0% SiC_p_ preform consisted entirely of fine particles. The image revealed a large number of particles homogeneously distributed throughout the preform, and the pores were divided and refined by the particles. In the c-25% SiC_p_ preform (Figure 2b), there were many fine particles and few coarse particles; the coarse particles were separately distributed with fine particles surrounding them. As the fraction of coarse particles in the bimodal SiC_p_ preform continued to increase (Figure 2c), the homogeneity of the particle distribution decreased. Coarse particles began to agglomerate in some areas of the cross section. There were large pores between adjacent coarse particles, and fine particles filled the pores. As shown in Figure 2d, the agglomeration of coarse particles became more significant in the c-67% SiC_p_ preform, with a large number of fine particles filling the pores between adjacent coarse particles. When the coarse particle fraction of the bimodal SiC_p_ preform was increased to 75%, the cross section (Figure 2e) showed a large number of coarse particles distributed throughout, while the number of fine particles was significantly reduced; the fine particles were insufficient to fill the pores between the coarse particles. In the c-100% SiC_p_ preform (Figure 2f), the preform consisted entirely of coarse particles. The pores between particles were large, and the size of individual pores increased significantly. Due to the large particle size, particle agglomeration was obvious in some areas of the preform, resulting in a significant decrease in pore distribution homogeneity.

### 3.2. Distribution of Areal Porosity of Preforms

**Figure 3:**
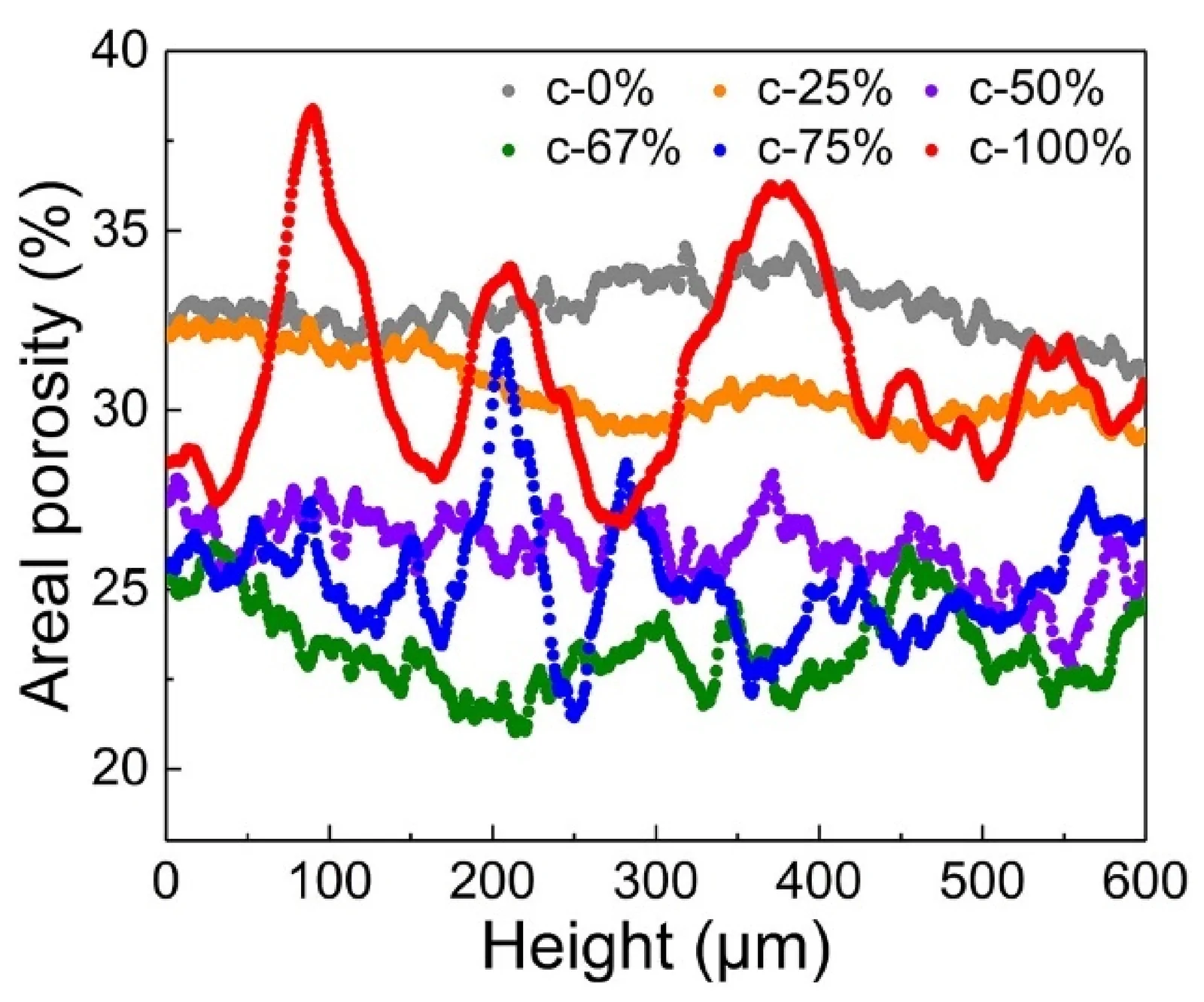
Areal porosity distributions of cross sections in bimodal SiC_p_ preforms.

The porosities of the bimodal SiC_p_ preforms were analyzed to assess the pore distribution. As shown in Table 1, the porosities of the bimodal SiC_p_ preforms measured with Archimedes’ method were very close to the average areal porosities determined by 3D μ-CT. When the coarse particle fraction in the bimodal SiC_p_ preforms increased from 0% to 67%, the average areal porosity gradually decreased from 32.79% to 23.36%, while as the coarse particle fraction increased from 67% to 100%, the average areal porosity gradually increased from 23.36% to 31.12%. The areal porosities of 600 cross sections along the *Z*-axis of the bimodal SiC_p_ preforms were measured, and the variations in the areal porosities are displayed in Figure 3. When the coarse particle fraction was low (0% and 25%), the variation range of the areal porosities was relatively small. As the coarse particle fraction increased, the variation range gradually widened.

**Figure 4:**
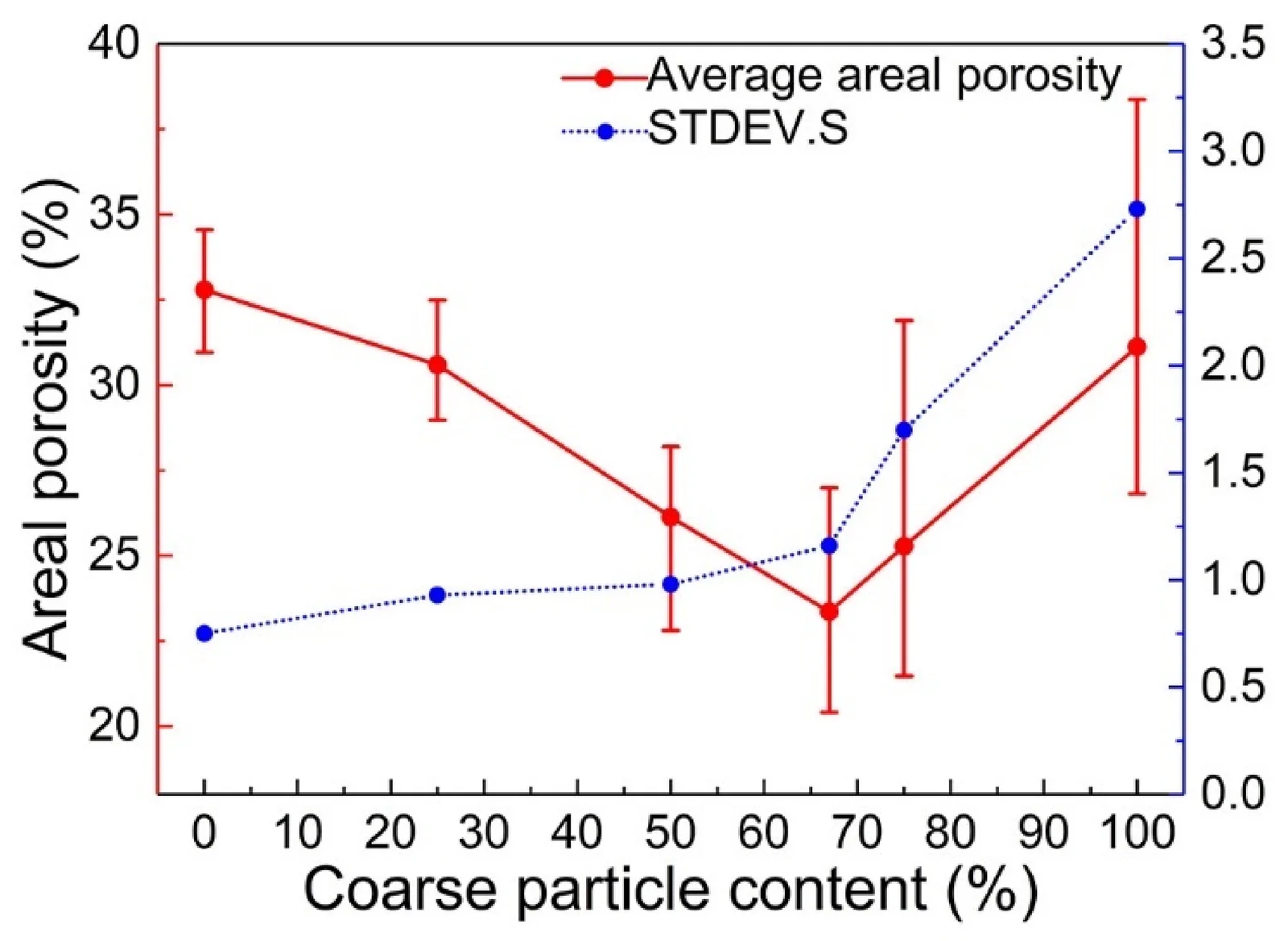
Average areal porosities and their STDEV.S values for bimodal SiC_p_ preforms.

The STDEV.S values (representing the statistical variation within each area) of the areal porosities of the bimodal SiC_p_ preforms were calculated and analyzed, and the results are presented in Table 2. According to the data in Table 1 and Table 2, the areal porosity distributions and their STDEV.S values for the bimodal SiC_p_ preforms were as shown in Figure 4. As the coarse particle fraction increased, the average areal porosities of the bimodal SiC_p_ preforms first decreased and then increased, and the average areal porosity was lowest when the coarse particle fraction was 67%. The STDEV.S values of the areal porosities continued to increase, indicating a continuous decline in the homogeneity of pore distributions.

### 3.3. Three-Dimensional Characteristics of Preforms

**Figure 5:**
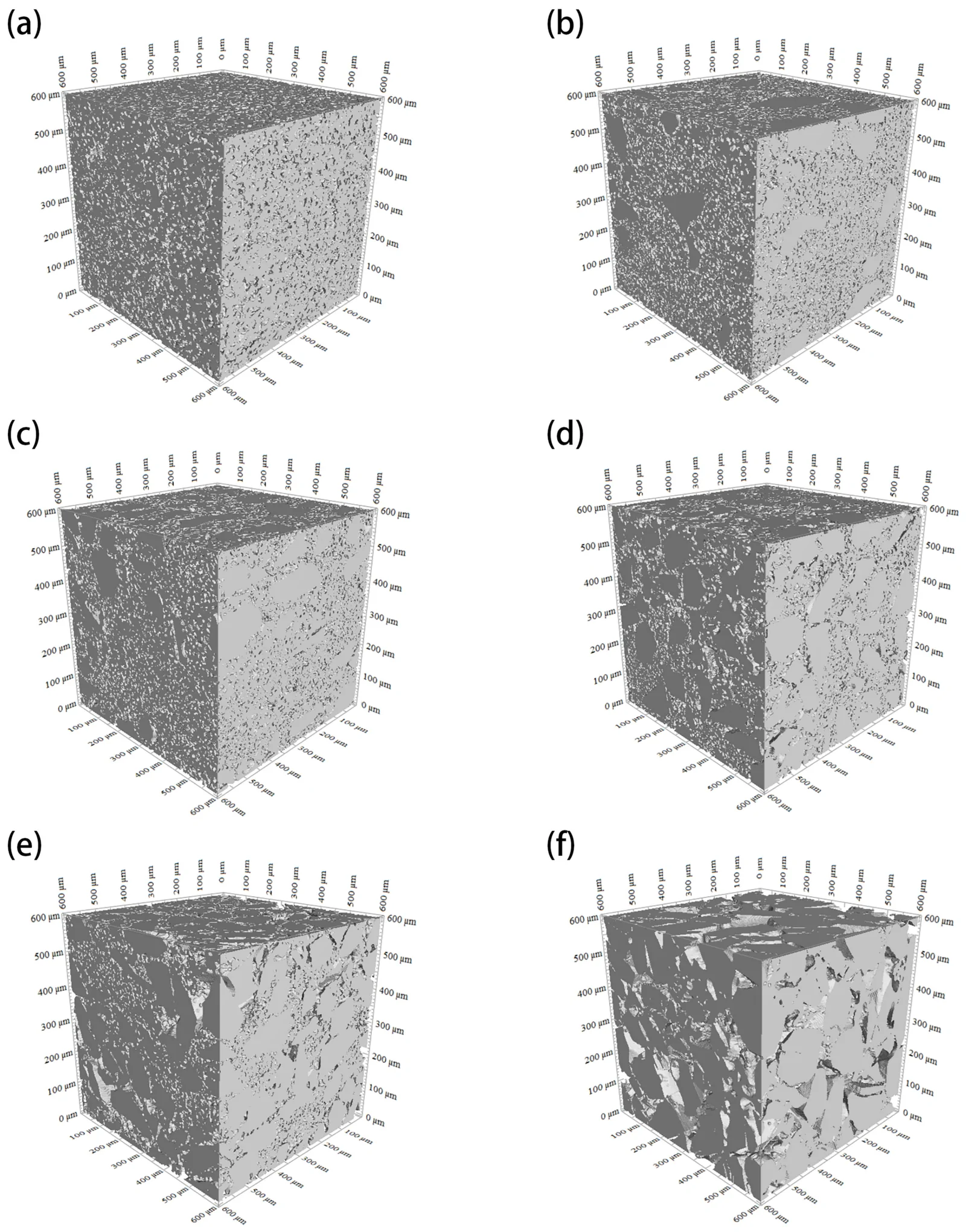
Three-dimensional morphologies of particle skeletons in bimodal SiC_p_ preforms: (**a**) c-0%, (**b**) c-25%, (**c**) c-50%, (**d**) c-67%, (**e**) c-75%, and (**f**) c-100%.

**Figure 6:**
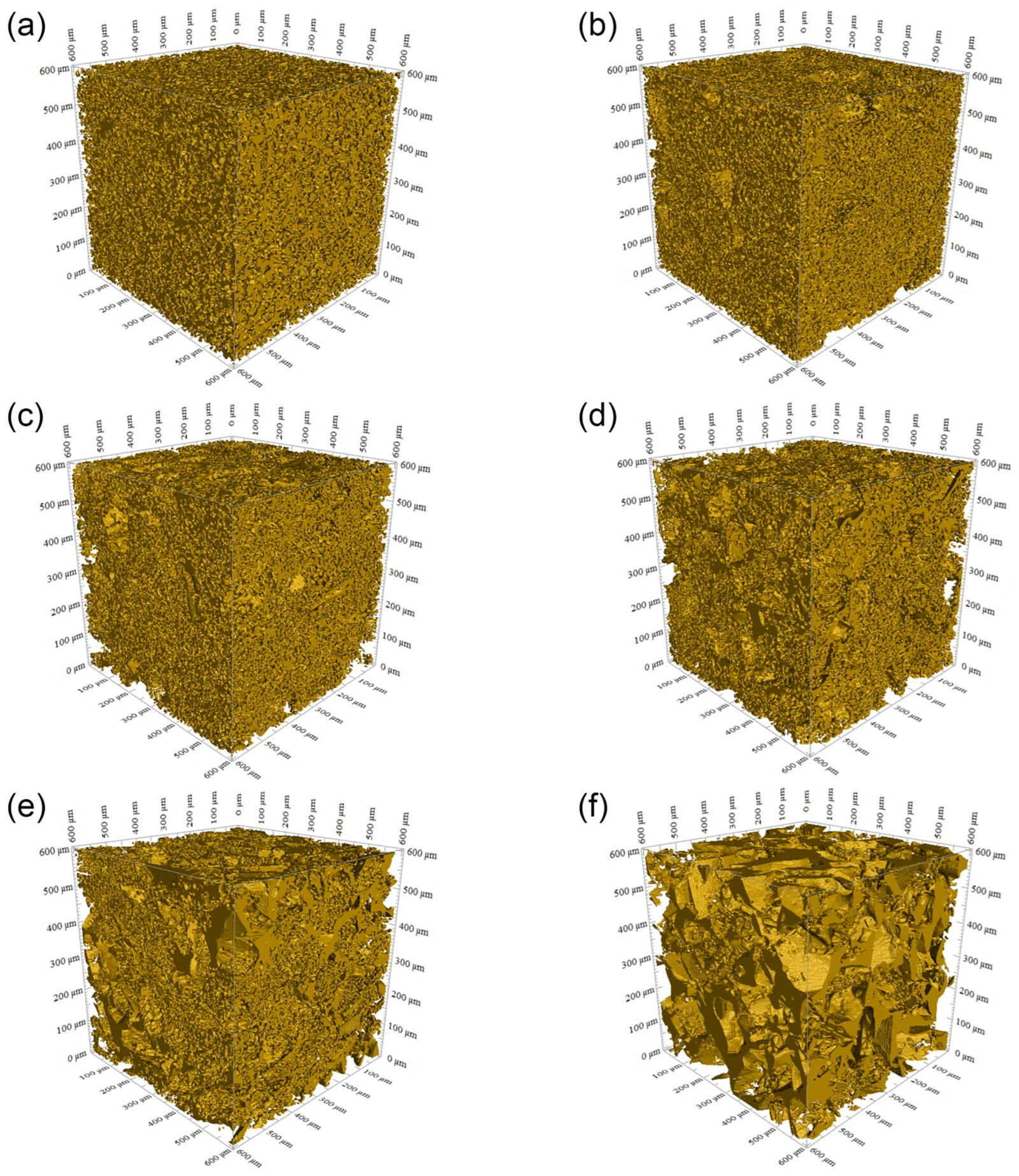
Three-dimensional morphologies of pores in bimodal SiC_p_ preforms: (**a**) c-0%, (**b**) c-25%, (**c**) c-50%, (**d**) c-67%, (**e**) c-75%, and (**f**) c-100%.

To further investigate the pore characteristics of the bimodal SiC_p_ preforms, the morphologies of their *Z*-axis cross sections were reconstructed to obtain the 3D morphologies of their particle skeletons (Figure 5) and pores (Figure 6). In the VOI, it can be clearly observed that the pores were highly interconnected, and the pore shapes became more angular and irregular with increasing coarse particle fraction in the preforms.

In studies on the pore characteristics of bimodal SiC_p_ preforms, the surface area per unit volume is a key factor. Molina [14,15] calculated the surface area per unit volume of particles in bimodal SiC_p_ preforms with empirical equations:

(1) $$S_{p}\;=\;\left(1-x_{c}\right)S_{\mathrm{fp}}\;+\;x_{c}S_{\mathrm{cp}}$$

(2) $$S_{\mathrm{fp}/\mathrm{cp}}=\rho_{p}S_{\mathrm{BET}}$$

where $S_{p}$ is the surface area per unit volume of particles in each bimodal SiC_p_ preform; $S_{\mathrm{fp}}$ and $S_{\mathrm{cp}}$ are the surface areas per unit volume of fine and coarse particles, respectively; $x_{c}$ is the fraction of coarse particles in the preform; $\rho_{p}$ is the density of SiC particles (3.21 g/cm^3^); and $S_{\mathrm{BET}}$ is the specific surface area of SiC particles measured from BET analysis using a specific surface area analyzer (V-Sorb 2800TP, Gold APP Instruments Corporation China (Beijing, China)). The specific surface areas of 20 and 170 μm SiC particles are 148.63 and 12.73 m^2^/kg, respectively.

Using Equation (2), the surface areas per unit volume of fine and coarse SiC particles were calculated to be 0.4771 and 0.0409 μm^−1^, respectively. The surface area per unit volume of particles in each bimodal SiC_p_ preform can be determined with Equation (1) and 3D μ-CT measurement. To analyze the accuracy of the surface areas per unit volume obtained from μ-CT measurement, validation was performed with BET analysis. Table 3 lists the calculated and measured surface area per unit volume of particles in each bimodal SiC_p_ preform. As can be seen, the surface areas gradually decreased with an increase in the coarse particle fraction.

**Figure 7:**
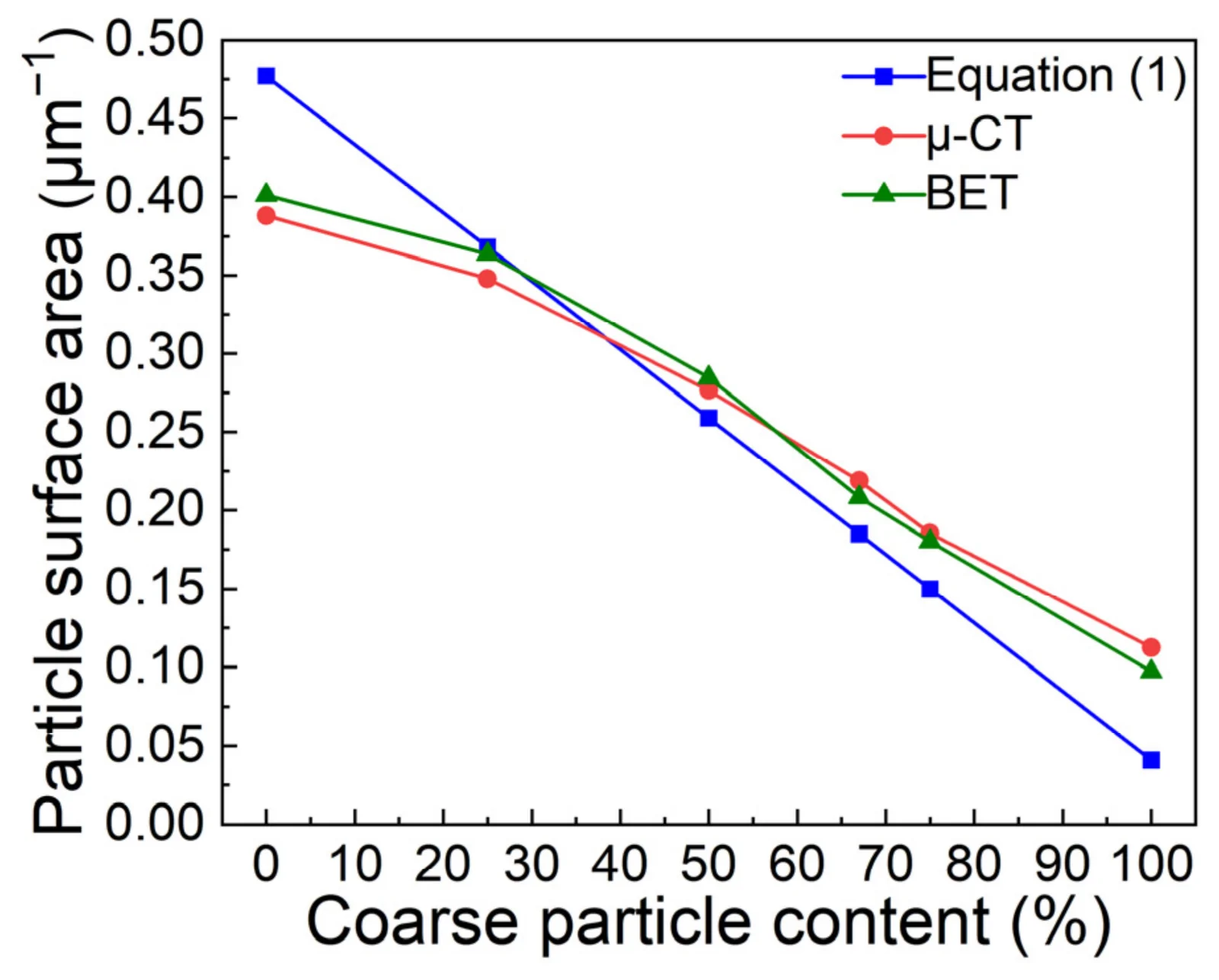
Curve of surface area per unit volume of particles in bimodal SiC_p_ preforms.

Although the calculated surface areas per unit volume of particles show a similar trend to the measured results, there are still differences in the values. Figure 7 shows the curve of the surface area per unit volume of particles in the bimodal SiC_p_ preforms. As depicted in the figure, the 3D μ-CT-measured surface area per unit volume of particles in the c-0% SiC_p_ preform was lower than the value calculated with Equation (1). The μ-CT-measured value for the c-25% preform was very close to the calculated one. However, when the coarse particle fraction increased to 50%, the measured value exceeded the calculated one. As the coarse particle fraction continued to increase, the measured values gradually deviated from the calculated values. The μ-CT-measured values were consistent with the BET results, confirming the accuracy of μ-CT measurements. The underlying mechanisms were further analyzed based on cross-sectional particle morphologies.

**Figure 8:**
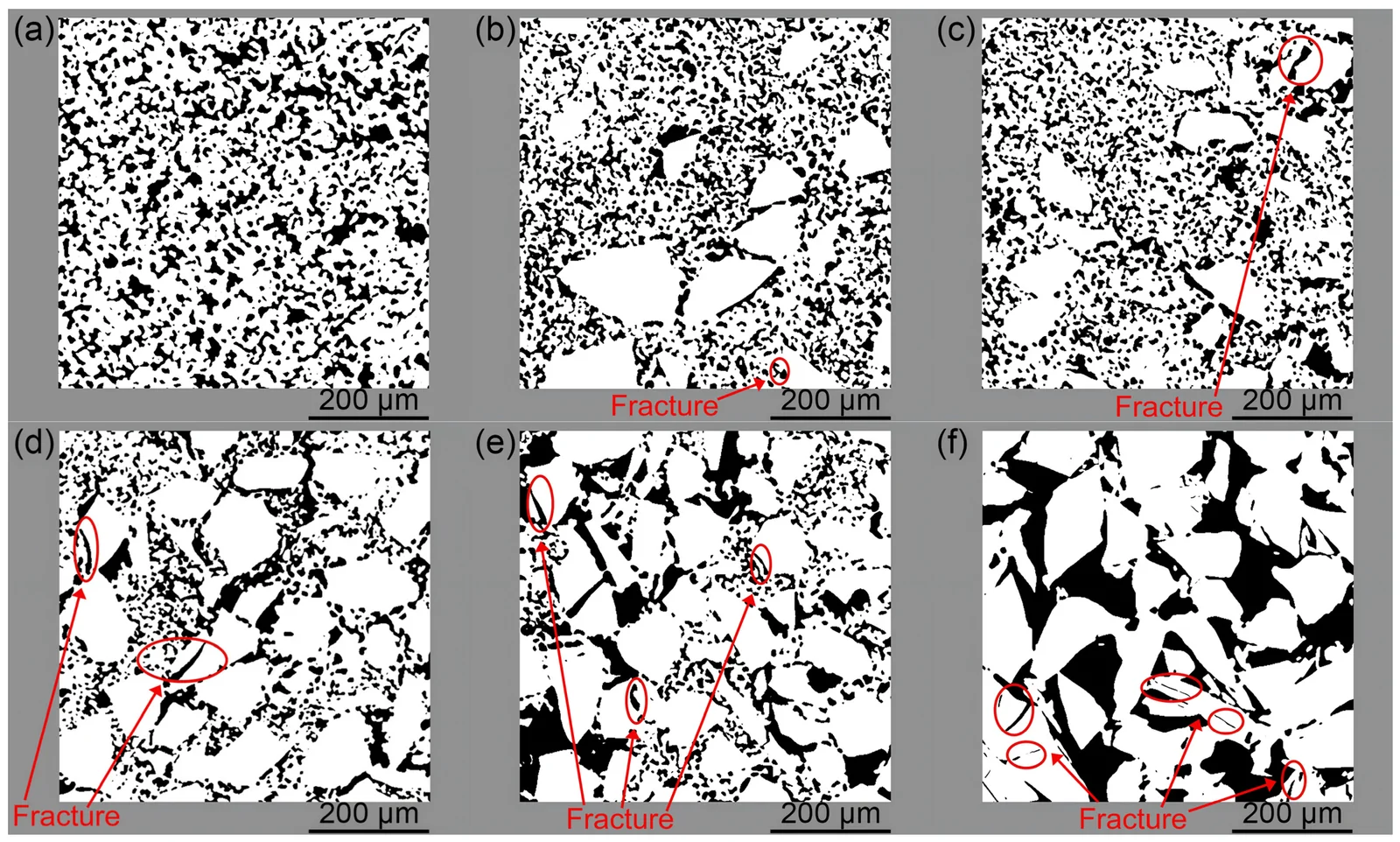
Particle morphologies of *Z*-axis cross sections in bimodal SiC_p_ preforms: (**a**) c-0%, (**b**) c-25%, (**c**) c-50%, (**d**) c-67%, (**e**) c-75%, and (**f**) c-100%.

Figure 8 displays the particle morphologies of *Z*-axis cross sections in the bimodal SiC_p_ preforms. As can be seen in Figure 8a, a large number of fine particles were homogeneously distributed throughout the cross section of the c-0% preform. These fine particles were in close contact with one another, which reduced the surface area per unit volume of particles in the preform. Consequently, the surface area per unit volume of particles measured by 3D μ-CT was smaller than the value calculated with Equation (1). In the c-25% preform (Figure 8b), the fine particles remained in close contact with one another, while the contact compactness had decreased compared to that in the c-0% preform. The results measured by μ-CT showed no significant difference from the values calculated using Equation (1). This was attributed to the fact that the coarse particles were prone to fracturing (as marked with red ellipses in the figure) under pressure during the compression molding process, resulting in the formation of fine particles. With increasing coarse particle fraction in the bimodal SiC_p_ preforms, the number of fractured coarse particles rose and the contact compactness of particles decreased, causing the results from μ-CT measurements to gradually exceed the values calculated with Equation (1). In the c-100% preform (Figure 8f), a large number of coarse particles within the cross section had fractured into fine particles, resulting in a significant increase in the surface area per unit volume of particles. Therefore, the measured values from μ-CT were notably higher than the ones calculated with Equation (1). In summary, the effect of contact compactness outweighed that of coarse particle fracture when the coarse particle fraction was less than 25%, resulting in the measured values from μ-CT being lower than the values calculated with Equation (1). Conversely, when the coarse particle fraction exceeded 25%, the effect of coarse particle fracture was much more significant, resulting in higher measured values. It can thus be seen that the empirical equation calculated the surface area per unit volume of particles by simply multiplying the surface area per unit volume of each particle with its fraction, whereas the μ-CT method accounted for the effects of contact compactness and fracture in coarse particles. Accordingly, the present comparison indicates that μ-CT provides a detailed description of the surface area per unit volume in these preforms.

### 3.4. Analysis of 3D Pore Networks of Preforms

**Figure 9:**
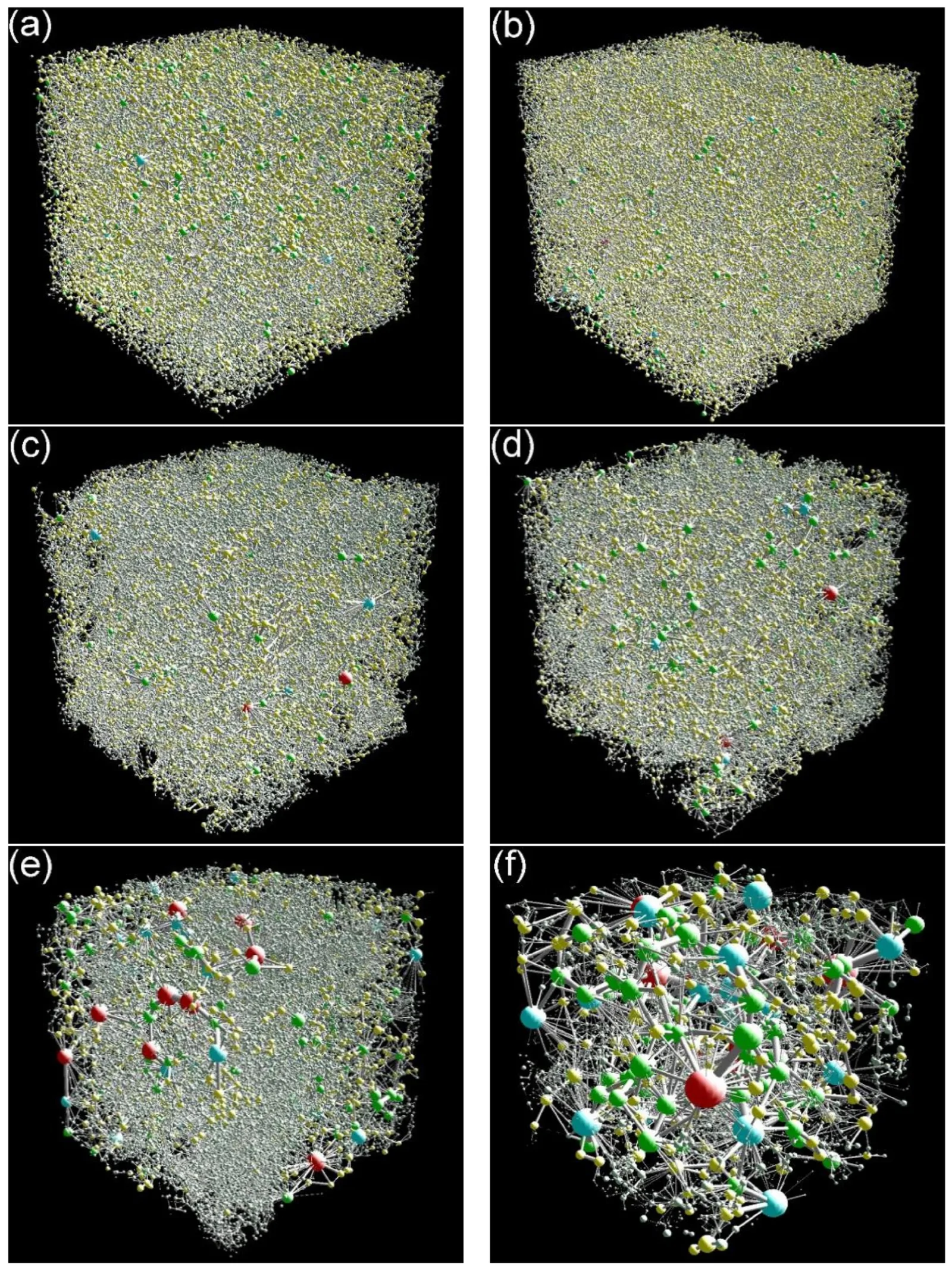
Three-dimensional pore networks of bimodal SiC_p_ preforms: (**a**) c-0%, (**b**) c-25%, (**c**) c-50%, (**d**) c-67%, (**e**) c-75%, and (**f**) c-100%. Pores and throats are represented by balls and spokes, respectively, and the balls with various colors correspond to pores of different radii.

Figure 9 presents 3D pore networks of bimodal SiC_p_ preforms with different coarse-to-fine particle ratios. As shown in Figure 9a,b, the 3D pore networks of the c-0% and c-25% preforms contained a large number of small-sized pores and throats. As the coarse particle fraction in the preform increased from 50% to 75% (Figure 9c–e), the total number of pores and throats gradually decreased, and the number of small-sized pores and throats also decreased, while the sizes of both pores and throats gradually increased. In the c-100% preform (Figure 9f), the number of pores and throats decreased significantly, and their maximum sizes increased markedly.

The numbers of pores and throats in the 3D pore networks of the bimodal SiC_p_ preforms were counted. The average values of pore characteristics such as the radius, volume and throat length for each pore and throat were calculated. Table 4 lists the 3D characteristics of pores and throats of bimodal SiC_p_ preforms.

**Figure 10:**
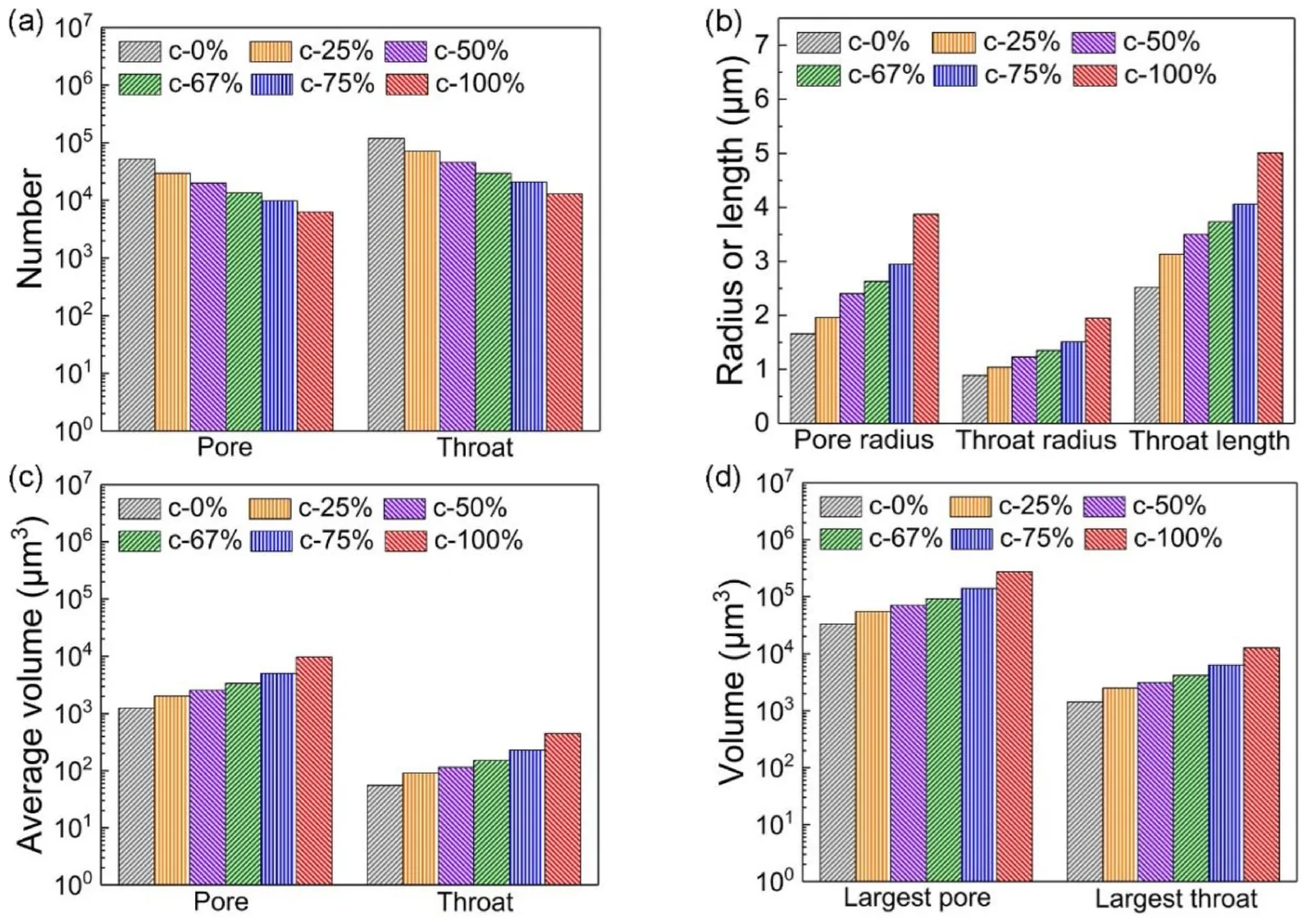
Main characteristics of 3D pore networks of bimodal SiC_p_ preforms: (**a**) numbers of pores and throats, (**b**) average radii and throat lengths, (**c**) average volumes of pores and throats, and (**d**) volumes of the largest pore and throat.

Figure 10 shows the main characteristics of the 3D pore networks of the bimodal SiC_p_ preforms. With increasing coarse particle fraction, the numbers of pores and throats in the preforms decreased significantly; the throat lengths and the average radii and volumes of pores and throats increased, as did the volumes of the largest pores and throats.

**Figure 11:**
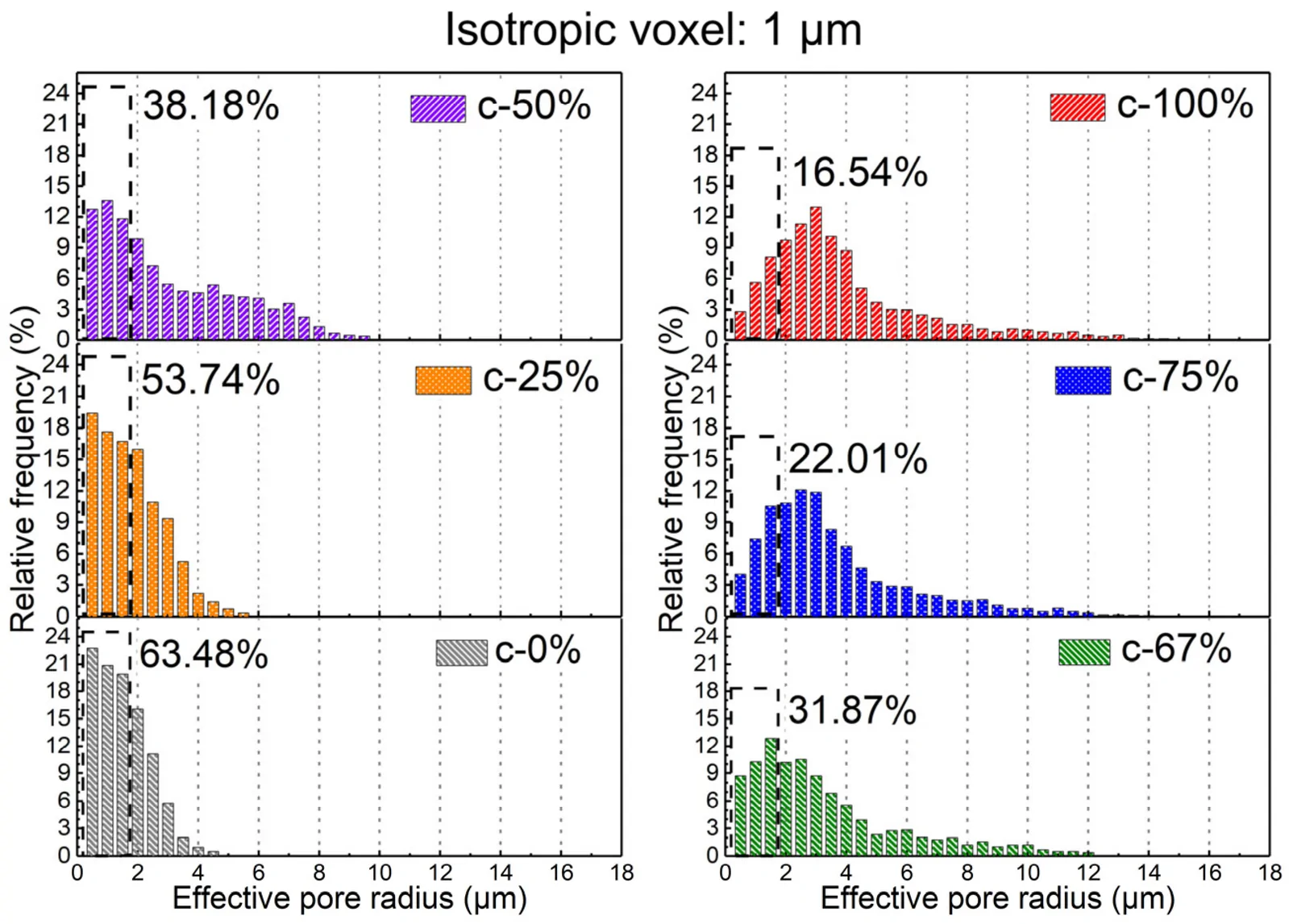
Distributions of pore radii of 3D pore networks in bimodal SiC_p_ preforms.

The distributions of pore radii of 3D pore networks in the bimodal SiC_p_ preforms are plotted in Figure 11. Since the c-0% preform had the smallest average pore radius (1.66 μm), pores with radii less than 1.66 μm were defined as small-sized pores. The relative frequencies of small-sized pores were calculated and compared. As shown in the figure, the relative frequencies of small-sized pores in the 3D pore networks decreased (dropping from 63.48% to 16.54%) with increasing coarse particle fraction in the preform.

**Figure 12:**
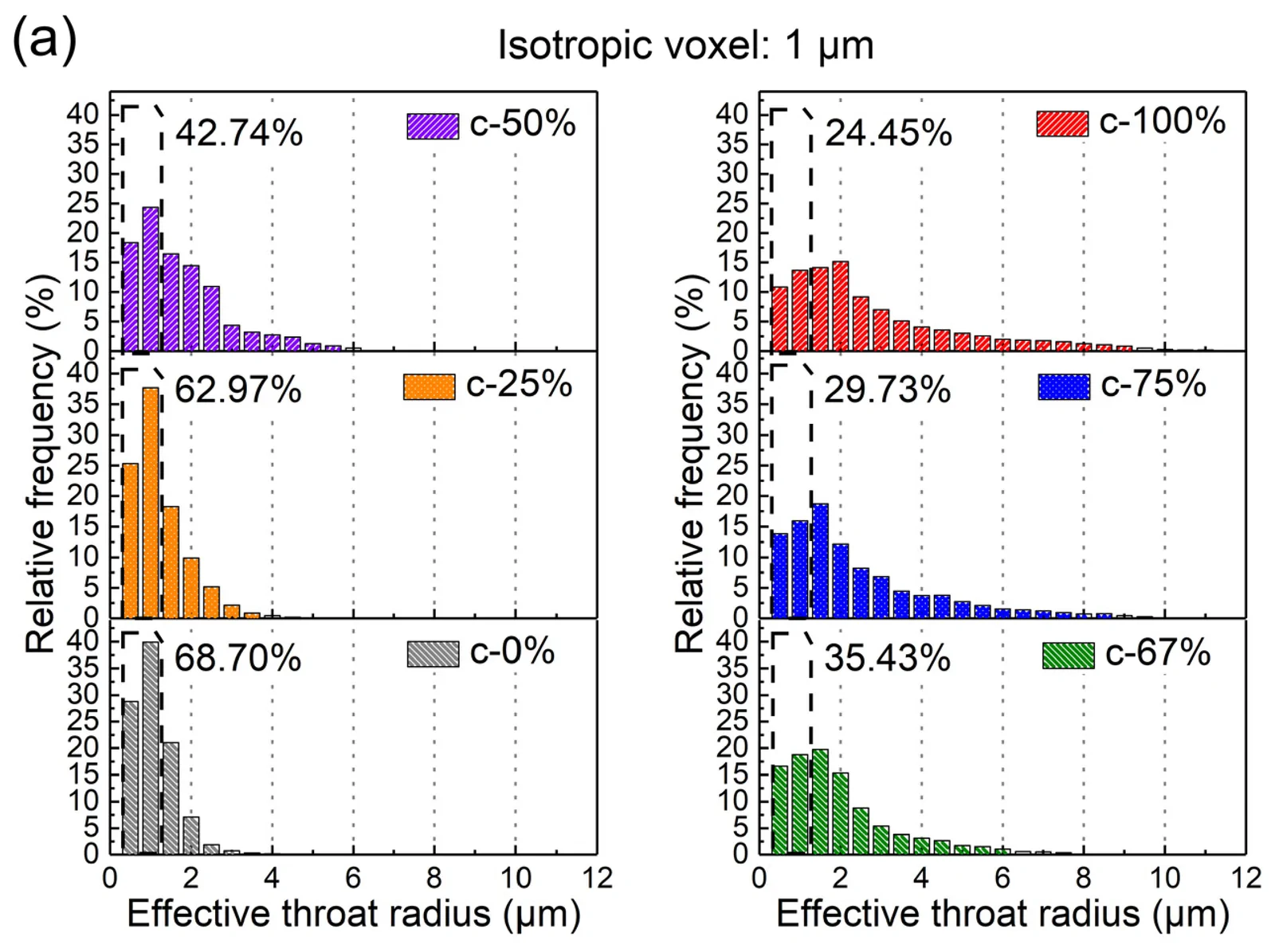

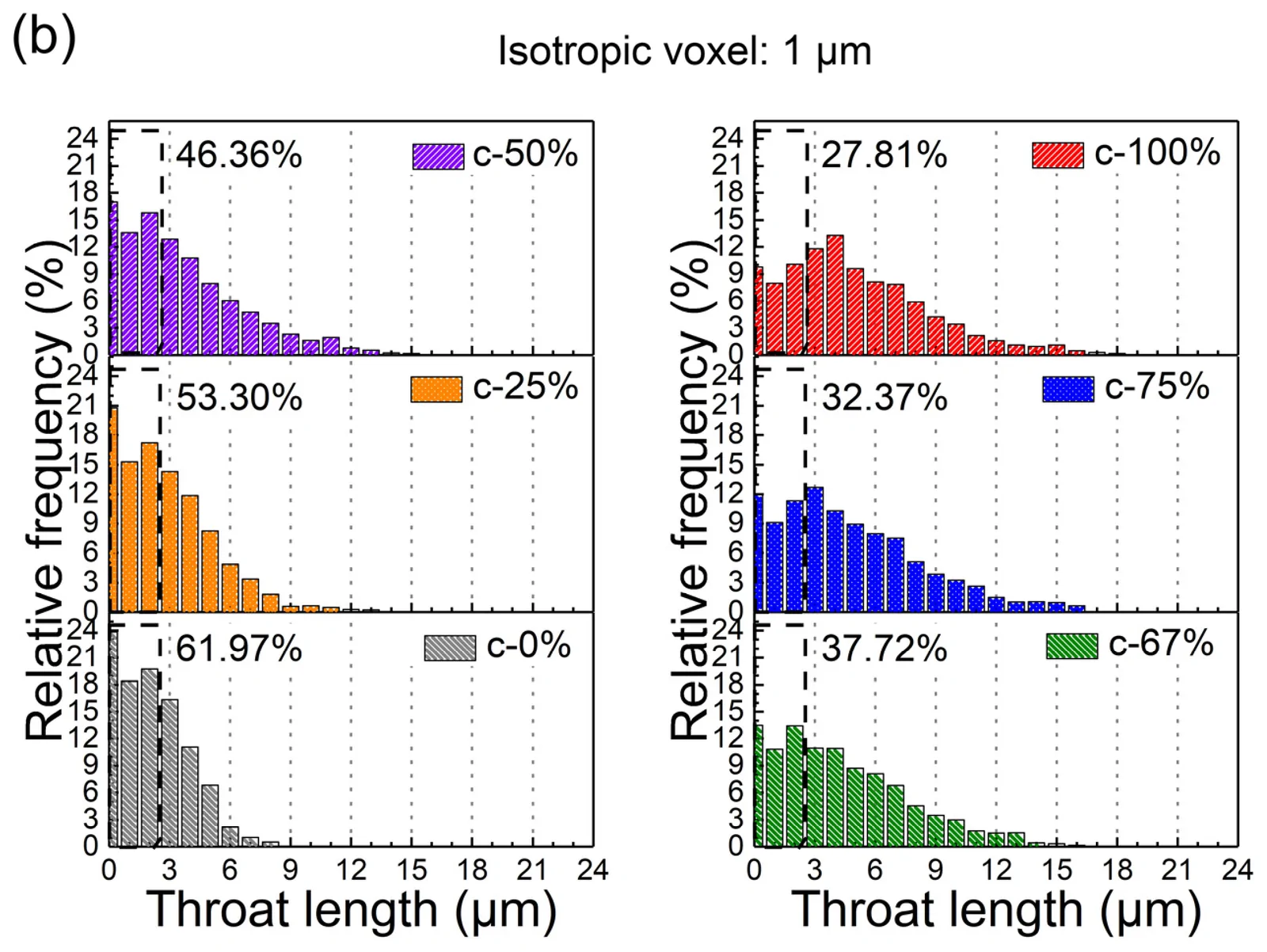
Distributions of throat radii and lengths of 3D pore networks in bimodal SiC_p_ preforms: (**a**) throat radius and (**b**) throat length.

Figure 12 shows the distributions of throat radii and lengths in the 3D pore networks in the bimodal SiC_p_ preforms. Taking the average throat radius (0.89 μm) and average throat length (2.52 μm) of the c-0% preform as the standard, throats with radii less than 0.89 μm were defined as small-sized throats and those with lengths less than 2.52 μm were defined as short throats. The relative frequencies of small-sized and short throats were statistically analyzed. The results shown in the figure indicate that as the coarse particle fraction of the preform increased, the relative frequencies of short and small-sized throats in the 3D pore networks gradually decreased.

## 4. Conclusions

Through 3D X-ray μ-CT, the 3D pore characteristics of bimodal SiC_p_ preforms with different ratios of coarse and fine particles were characterized and analyzed, and the results were compared with those from Molina’s studies. The main conclusions are as follows:

The average areal porosities of the bimodal SiC_p_ preforms initially decreased then increased with rising coarse particle fraction, reaching a minimum of 23.36% at a 67% coarse fraction. However, increasing the coarse fraction elevated the STDEV.S value of areal porosity and reduced the distribution homogeneity because coarse particle agglomeration prevented fine particles from fully filling inter-particle pores.

A discrepancy emerged between empirical calculations and 3D μ-CT measurements of the particle surface area per unit volume: below a 25% coarse fraction, particle contact compactness caused lower measured values; above 25%, particle fracturing during molding caused higher measured values. The 3D μ-CT measurements reflect both particle contact and fracturing behaviors, delivering a high-resolution structural representation method.

As the coarse particle fraction increased, the 3D pore networks of the bimodal SiC_p_ preforms showed a clear trend toward coarsening: the pore and throat numbers decreased significantly, while their average dimensions and volumes increased. At the same time, the relative frequencies of small-sized pores and throats gradually decreased.

## Figures and Tables

**Table 1:** Porosities of bimodal SiC_p_ preforms.

SiC_p_ Preform	c-0%	c-25%	c-50%	c-67%	c-75%	c-100%
Model volume	600 × 600 × 600 μm^3^					
Porosity (Archimedes)	32.8% (±0.35%)	30.6% (±0.35%)	26.2% (±0.25%)	23.4% (±0.21%)	25.3% (±0.25%)	31.2% (±0.39%)
Average areal porosity (μ-CT)	32.79% (±0.40%)	30.60% (±0.38%)	26.13% (±0.20%)	23.36% (±0.23%)	25.27% (±0.31%)	31.12% (±0.43%)
Minimum areal porosity	30.96% (±0.41%)	28.98% (±0.37%)	22.80% (±0.19%)	21.01% (±0.19%)	21.45% (±0.4%)	26.81% (±0.46%)
Maximum areal porosity	34.55% (±0.49%)	32.49% (±0.42%)	28.20% (±0.27%)	26.24% (±0.28%)	31.87% (±0.44%)	38.37% (±0.50%)

**Table 2:** STDEV.S values of areal porosities of bimodal SiC_p_ preforms.

SiC_p_ Preform (Three Samples)	c-0%	c-25%	c-50%	c-67%	c-75%	c-100%
STDEV.S	0.75 (0.73/0.76)	0.93 (0.90/0.95)	0.98 (1.01/0.96)	1.16 (1.12/1.19)	1.70 (1.66/1.73)	2.73 (2.68/2.77)

**Table 3:** Surface area per unit volume of particles in bimodal SiC_p_ preforms.

SiC_p_ Preform	c-0%	c-25%	c-50%	c-67%	c-75%	c-100%
Particle surface area (Equation (1)) (μm^−1^)	0.4771	0.3680	0.2589	0.1848	0.1499	0.0409
Particle surface area (μ-CT) (μm^−1^)	0.3881	0.3479	0.2765	0.2187	0.1854	0.1125
Particle surface area (BET) (μm^−1^)	0.4013	0.3634	0.2846	0.2083	0.1801	0.0972

**Table 4:** Three-dimensional characteristics of pores and throats of bimodal SiC_p_ preforms.

SiC_p_ Preform (Three Samples)	c-0%	c-25%	c-50%	c-67%	c-75%	c-100%
Number of pores	51,596 (52,473/50,668)	29,450 (29,862/29,037)	19,963 (20,302/19,604)	13,560 (13,776/13,329)	9836 (10,042/9649)	6279 (6417/6166)
Number of throats	118,671 (120,095/117,128)	71,457 (72,743/70,109)	45,615 (46,572/44,657)	29,530 (30,177/28,880)	20,773 (21,146/20,297)	12,877 (13,083/12,581)
Average pore radius (μm)	1.66 (1.65/1.68)	1.96 (1.94/1.98)	2.40 (2.36/2.43)	2.63 (2.59/2.67)	2.95 (2.90/3.01)	3.87 (3.81/3.96)
Average throat radius (μm)	0.89 (0.88/0.90)	1.04 (1.03/1.06)	1.23 (1.21/1.25)	1.35 (1.32/1.38)	1.51 (1.48/1.55)	1.95 (1.91/1.99)
Average throat length (μm)	2.52 (2.49/2.54)	3.13 (3.08/3.17)	3.50 (3.44/3.55)	3.73 (3.66/3.80)	4.06 (3.96/4.15)	5.01 (4.88/5.12)
Average pore volume (μm^3^)	1243.99 (1230.31/1257.66)	2022.51 (1986.09/2060.93)	2544.31 (2511.23/2582.47)	3355.86 (3312.23/3406.20)	5042.29 (4951.53/5122.97)	9770.38 (9584.75/9936.46)
Average throat volume (μm^3^)	55.32 (54.21/56.30)	90.76 (89.58/91.84)	114.62 (112.33/116.92)	151.98 (148.46/154.57)	229.39 (225.95/235.12)	446.92 (440.22/455.09)
Volume of the largest pore (μm^3^)	32,993 (32,465/33,454)	54,932 (54,053/55,865)	70,121 (68,997/71,242)	91,210 (89,568/93,307)	139,118 (136,057/141,622)	273,861 (267,288/279,063)
Volume of the largest throat (μm^3^)	1416 (1396/1439)	2506 (2455/2544)	3135 (3066/3194)	4171 (4105/4223)	6367 (6240/6493)	12,562 (12,311/12,750)

## Data Availability

The original contributions presented in this study are included in the article. Further inquiries can be directed to the corresponding author.

## References

[1] Zhou C., Tang Z.Y., Kong X.Q., Zhou Y.X., Liao M.Q., Qian J.R., Liu C.X., Song Y.Z., Liu Z.K., Fan L.L. (2024). High-performance zero thermal expansion in Al metal matrix composites. Acta Mater..

[2] Qian X., Sun S., Ye J., Zhang C., Ru H. (2023). Continuous SiC Skeleton-Reinforced Reaction-Bonded Boron Carbide Composites with High Flexural Strength. Materials.

[3] Li W., Zhang G., Cui C., Bao J., Guo C., Xu C., Zhang W., Zhu W. (2022). Structure Evolution and Properties Modification for Reaction-Bonded Silicon Carbide. Materials.

[4] Wang C.J., Wang C., Li Z.H., He Y.H., Zhang Z. (2024). Effect of vacuum infiltration temperature on the microstructure and tribological properties of honeycomb-filled tube-reinforced aluminum matrix composites. Mater. Chem. Phys..

[5] Naikade M., Hain C., Kastelik K., Parrilli A., Graule T., Weber L., Ortona A. (2023). Liquid metal infiltration of silicon based alloys into porous carbonaceous materials Part-III: Experimental verification of conversion products and infiltration depth by infiltration of Si-Zr alloy into mixed SiC/graphite preforms. J. Eur. Ceram. Soc..

[6] Song M.X., Peng K. (2023). Pre-oxidization of SiC on interfacial structure and mechanical properties of SiCp/Al composites prepared by vacuum-pressure infiltration. J. Mater. Res. Technol..

[7] Bhatti T.M., Wang Y.W., Baig M.M.A.B., Zhou Z.F., Hussain A., Jamal S., Shehzadi F. (2025). Optimized thermo-mechanical behavior of SiCp/Al composites through modified preform preparation process. Mater. Charact..

[8] Guo W.M., Xiao H.N., Yao X.H., Liu J.X., Liang J.J., Gao P.Z., Zeng G.M. (2016). Tuning pore structure of corrosion resistant solid-state-sintered SiC porous ceramics by particle size distribution and phase transformation. Mater. Des..

[9] Jin Y.C., Zhang B., Zhang H.Q., Zhong Z.X., Wang Y., Ye F., Liu Q. (2021). Effects of skeleton pore size on the microstructure and electromagnetic absorbing property of the SiC nanowires/SiC composites. Mater. Lett..

[10] Wang Z.P., Zhao K., Liu J.L., An L.N. (2025). Enhanced mechanical properties of aluminum matrix composite reinforced by bimodal-sized (submicron + nano) particles. J. Alloys Compd..

[11] Feng M.Z., Wang Z.W., Wang W.Q., Tian J.D., Jiang Y.L., Xing B.H., Zhao Z. (2023). Effect of bimodal particle size distribution on the performance of SiC slurry for maskless vat photopolymerization. J. Eur. Ceram. Soc..

[12] Sadeghi B., Cavaliere P., Laska A., Perrone A., Blasi G., Gopinathan A., Shamanian M., Ashrafizadeh F. (2023). Effect of processing parameters on the cyclic behaviour of aluminium friction stir welded to spark plasma sintered aluminium matrix composites with bimodal micro-and nano-sized reinforcing alumina particles. Mater. Charact..

[13] Molina J.M., Piñero E., Narciso J., García-Cordovilla C., Louis E. (2005). Liquid metal infiltration into ceramic particle preforms with bimodal size distributions. Curr. Opin. Solid State Mater. Sci..

[14] Molina J.M., Saravanan R.A., Arpón R., García-Cordovilla C., Louis E., Narciso J. (2002). Pressure infiltration of liquid aluminium into packed SiC particulate with a bimodal size distribution. Acta Mater..

[15] Molina J.M., Narciso J., Weber L., Mortensen A., Louis E. (2008). Thermal conductivity of Al-SiC composites with monomodal and bimodal particle size distribution. Mater. Sci. Eng. A.

[16] Molina-Jordá J.M. (2015). Design of composites for thermal management: Aluminum reinforced with diamond-containing bimodal particle mixtures. Compos. Part A.

[17] Manes A., Giglio M. (2018). Microstructural numerical modeling of Al_2_O_3_/Ti composite. Procedia Struct. Integr..

[18] Mitchell I.T., Martini F., Stephens G.F., Middleburgh S.C., Vidal F.P. (2026). Assessing manufacturing defects in ceramic composites with both simulated and experimental synchrotron computed tomography. Prog. Nucl. Energy.

[19] Agrawal A.K., Gupta C., Singh B., Kashyap Y., Shukla M. (2024). Quantitative phase contrast X-ray tomography of aluminium metal matrix composite. Appl. Radiat. Isot..

[20] Luan X.G., Li Y., Hu C., Wu Y., Sun G.D., Zhao D.L., Cheng L.F. (2025). Effect of preform structures on creep properties for SiCf/SiC composites at 1300 °C in wet oxygen. Ceram. Int..

[21] Lu S., Zhu Q.Y., Zhao M., Zhuang Y., Liu S. (2026). Determination method of pore parameters in porous media for evaporation process based on pore network model and fractal theory. Int. J. Therm. Sci..

[22] Filimonov S.A., Pryazhnikov A.I., Guzei D.V., Minakov A.V. (2026). Development and testing of a new pore network algorithm for modeling flows of power law fluids in porous media. Adv. Eng. Softw..

[23] Yan L., Zhang X.W., Liu X.Y., Wang Q.Z., Wei G., Li X.M., Wang G. (2026). Multi-scale simulation of pore structure and permeability of brittle coral gravel using X-ray computerized tomography and pore network modeling. Appl. Ocean Res..

[24] Garcia S.G., Ling Y. (2026). Characterizing drop morphology evolution in aerodynamic breakup with topological skeleton. Comput. Fluids.

[25] Barnhill D., Yoshida R., Miura K. (2025). Maximum inscribed and minimum enclosing tropical balls of tropical polytopes and applications to volume estimation and uniform sampling. Comput. Geom.-Theory Appl..

[26] Gerke K.M., Sizonenko T.O., Karsanina M.V., Lavrukhin E.V., Abashkin V.V., Korost D.V. (2020). Improving watershed-based pore-network extraction method using maximum inscribed ball pore-body positioning. Adv. Water Resour..

[27] Liu R.Z., Xu H. (2026). Study on pore characteristics of irregular and regular particle preforms and their effects on molten aluminum flow. Mater. Today Commun..

[28] Liu R.Z., Zhao H.D., Xie B. (2020). Calculation of Infiltration of AlSi12 Alloys into Si Porous Preforms with General and Modified Infiltration Equations. Transp. Porous Media.

